# Asymmetric Construction of All-Carbon Quaternary Stereocenters by Chiral-Auxiliary-Mediated Claisen Rearrangement and Total Synthesis of (+)-Bakuchiol

**DOI:** 10.3390/molecules171113330

**Published:** 2012-11-08

**Authors:** Ken-ichi Takao, Shu Sakamoto, Marianne Ayaka Touati, Yusuke Kusakawa, Kin-ichi Tadano

**Affiliations:** Department of Applied Chemistry, Keio University, Hiyoshi, Kohoku-ku, Yokohama 223-8522, Japan

**Keywords:** Claisen rearrangement, chiral auxiliary, camphorsultam, quaternary stereocenter, total synthesis

## Abstract

An asymmetric Claisen rearrangement using Oppolzer’s camphorsultam was developed. Under thermal conditions, a geraniol-derived substrate underwent the rearrangement with good stereoselectivity. The absolute configuration of the newly formed all-carbon quaternary stereocenter was confirmed by the total synthesis of (+)-bakuchiol from the rearrangement product.

## 1. Introduction

**Figure 1:**
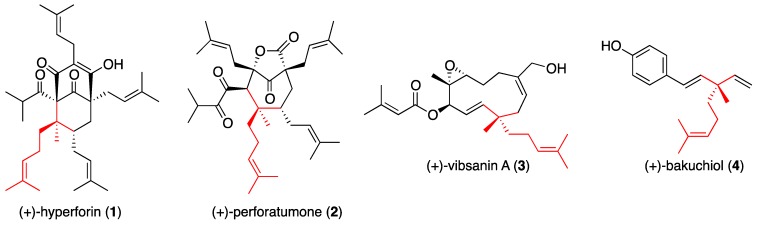
Structures of (+)-hyperforin, (+)-perforatumone, (+)-vibsanin A, and (+)-bakuchiol.

The construction of asymmetric quaternary stereocenters remains a challenge in organic synthesis [1,2,3]. All-carbon quaternary stereocenters are found in a wide range of complex natural products which share such a structural motif, including (+)-hyperforin (**1**) [4], (+)-perforatumone (**2**) [5,6], (+)-vibsanin A (**3**) [7], and (+)-bakuchiol (**4**) [8,9,10,11,12,13] (Figure 1). To achieve the total synthesis of these natural products, a practical method for constructing the quaternary stereocenter is necessary. We focused on the Claisen rearrangement as an approach to this challenge. The [3,3]-sigmatropic rearrangement of allyl vinyl ethers, that is, the Claisen rearrangement, is among the most useful tools for forming carbon-carbon bonds and its asymmetric variants have been well studied [14,15]. Herein, we describe a new method for the asymmetric construction of an all-carbon quaternary stereocenter by a chiral-auxiliary-mediated Claisen rearrangement.

## 2. Results and Discussion

**Scheme 1:**
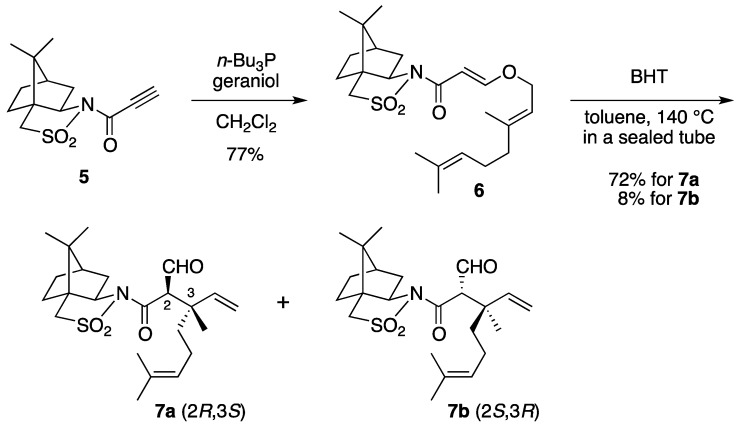
Claisen rearrangement of geraniol-derived substrate **6**.

Oppolzer’s camphorsultam was used as a chiral auxiliary for the asymmetric Claisen rearrangement. We designed a novel substrate, a β-(allyloxy)acrylate derivative bearing the camphorsultam. Accordingly, *N*-propioloyl camphorsultam **5** was prepared by our previously reported procedure (Scheme 1) [16,17,18]. The oxy-Michael addition of geraniol to **5** in the presence of a catalytic amount of tributylphosphine gave adduct **6** with complete *E*-stereoselectivity [19]. A toluene solution of **6** in the presence of butylated hydroxytoluene (BHT) used as a polymerization inhibitor was heated in a sealed tube at 140 °C to provide mainly the (2*R*,3*S*)-isomer **7a** as the rearrangement product in 72% yield, securing the two contiguous stereocenters including the quaternary carbon. The minor (2*S*,3*R*)-isomer **7b** (8%) was easily separated from **7a** by column chromatography on silica gel [20].

**Scheme 2:**
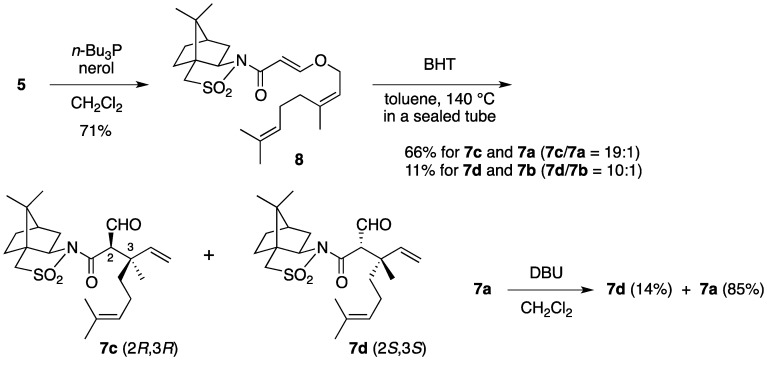
Claisen rearrangement of nerol-derived substrate **8**.

By using a similar procedure, nerol-derived substrate **8** was prepared from **5** and nerol (Scheme 2). The Claisen rearrangement of **8** afforded (2*R*,3*R*)-isomer **7c** and (2*S*,3*S*)-isomer **7d**, accompanied by a small amount of **7a** and **7b**, respectively. Compared with the case of **6**, however, lower stereoselectivity was observed. Brief exposure of **7a** to base caused epimerization at C-2 to produce isomer **7d**, indicating that the quaternary stereocenter in nerol-derived rearrangement product **7c** has stereochemistry opposite to that in **7a**.

**Scheme 3:**
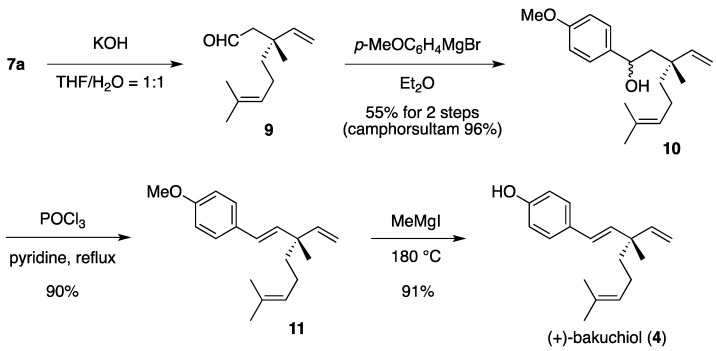
Determination of the stereochemistry at C-3 in **7a** and total synthesis of (+)-bakuchiol.

The stereochemistry of the newly formed quaternary stereocenter (C-3) in **7a** was determined by the total synthesis of (+)-bakuchiol (**4**), a major component of the Indian medicinal plant *Psoralea corylifolia* Linn [8]. Base hydrolysis of **7a** followed by decarboxylation provided enantiomerically pure aldehyde **9**, and the chiral auxiliary was recovered (Scheme 3). Treatment of **9** with *p*-MeOC_6_H_4_MgBr afforded alcohol **10**, which was subjected to dehydration using phosphoryl chloride to afford bakuchiol methyl ether **11** [21]. By comparing the optical rotation of synthetic **11** {[α]_D_^25^ + 28.4 (*c* 0.855, CHCl_3_)} with that reported for the authentic sample {lit. [α]_D_^29^ + 31.2 (*c* 1.45, CHCl_3_)} [9], the absolute configuration of the quaternary stereocenter in **7a** was assigned as (*S*). According to a known procedure [22], demethylation of **11** finally provided (+)-bakuchiol (**4**), which was identical to the natural product in all respects.

**Scheme 4:**
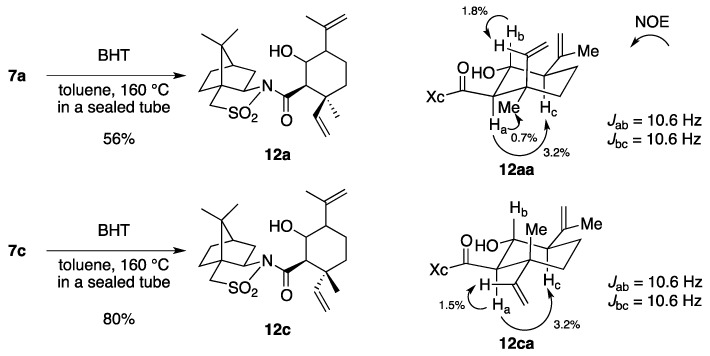
Determination of the stereochemistry at C-2 in **7a** and **7c**.

To determine the configuration at C-2, rearrangement product **7a** was heated at 160 °C (Scheme 4). The intramolecular carbonyl–ene reaction proceeded to provide cyclized **12a** as a mixture of four diastereomers (dr = 3:2:2:1). Similarly, **7c** was converted into **12c** (dr = 9:8:2:1). Through NOE experiments on the isolated major diastereomers **12aa** and **12ca**, the stereochemistry at the C-2 in **7a** and **7c** was assigned as (*R*). Therefore, the configurations of all stereocenters in the rearrangement products **7a**–**d** were unambiguously assigned.

**Scheme 5:**
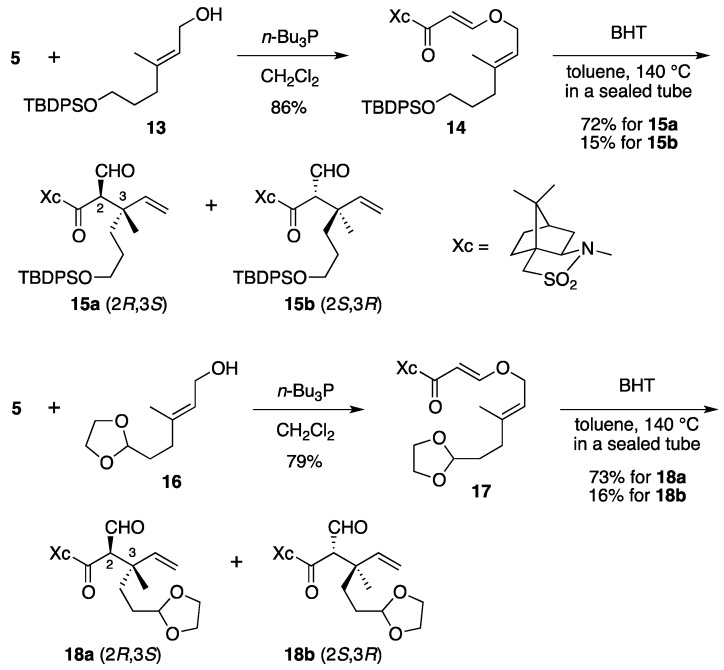
Claisen rearrangements of **14** and **17**.

To expand the scope of this reaction, (*E,E*)-β-(allyloxy)acrylate substrates **14** and **17** were synthesized by oxy-Michael addition of allylic alcohols **13** and **16** [23] to **5** (Scheme 5). In both cases, the Claisen rearrangements of **14** and **17** afforded (2*R*,3*S*)-isomers **15a** and **18a** preferentially, with good stereoselectivity in more than 70% yield, similarly to the reaction of **6**.

**Scheme 6:**
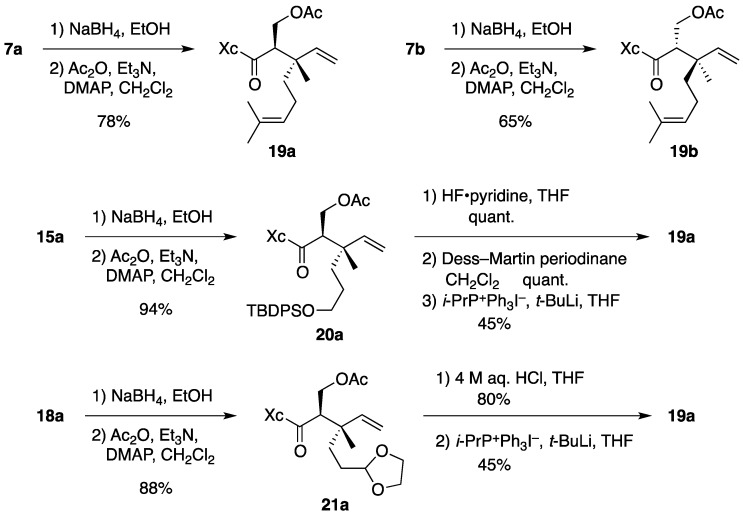
Determination of the stereochemistry at C-2 and C-3 in **15a** and **18a**.

The vicinal stereocenters in **15a** and **18a** were assigned by chemical transformation (Scheme 6). Chemoselective reduction of **7a**, followed by acetylation of the resulting alcohol, provided acetate **19a**. The spectroscopic data (^1^H- and ^13^C-NMR) of **19a** were distinguishable from those of **19b** derived from **7b**. On the other hand, **15a** was converted into acetate **20a**. Desilylation of **20a**, oxidation of the resulting alcohol to aldehyde, and subsequent Wittig olefination afforded **19a** whose NMR spectra matched those of **19a** derived from **7a**. Compound **18a** was also converted into **19a** via acetate **21a**. Therefore, the configuration of the vicinal stereocenters at the C-2 and C-3 in **15a** and **18a** coincides with that of **7a**.

**Scheme 7:**
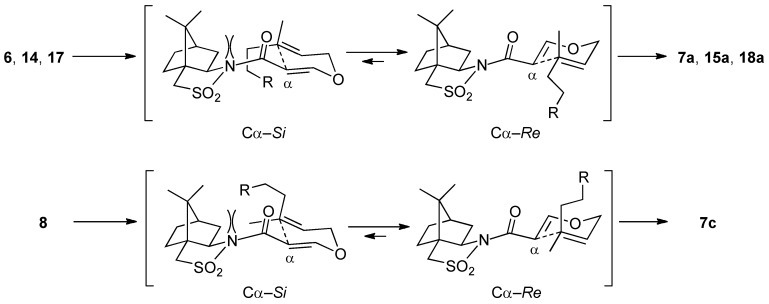
Plausible transition states for the Claisen rearrangements of **6**, **14**, **17**, and **8**.

The stereochemical outcomes observed in the reactions of **6**, **14**, **17**, and **8** can be explained by the transition states depicted in Scheme 7. 

In the more favorable conformation of **6**, **14**, and **17**, the carbonyl group is directed *anti* to the sulfonyl group and adopts an *s-cis* conformation with respect to the α,β-unsaturated bond [24]. The rearrangement proceeds predominantly from the Cα-*Re*-face through a six-membered chair-like transition state to avoid the steric repulsion that would be encountered along the Cα-*Si*-face path. As a result, **7a**, **15a**, and **18a** were obtained as the major isomers. Also nerol-derived substrate **8** rearranges through the same Cα-*Re*-face path to produce **7c**. In this case, the bulky homoprenyl group takes an axial orientation, which causes a decrease of the stereoselectivity.

## 3. Experimental

### General

Melting points are uncorrected. Specific rotations were measured in a 100 mm cell. ^1^H-NMR spectra were recorded at 500 MHz with tetramethylsilane as an internal standard on a JEOL JNM-ECA500 spectrometer. ^13^C-NMR spectra were recorded at 125 MHz. All spectra were recorded in CDCl_3_. High-resolution mass spectra (HRMS) were measured in EI mode (70 eV) on a JEOL JMS-GCmate spectrometer. Thin-layer chromatography (TLC) was performed on Merck Kieselgel 60 F_254_ plates. The crude reaction mixtures and extracted materials were purified by column chromatography on Silica gel 60 (Merck) or Wakogel C-300 (Wako). Unless otherwise noted, reactions were carried out at room temperature. Combined organic extracts were dried over anhydrous Na_2_SO_4_. Solvents were removed from the reaction mixture and the combined organic extracts by concentration under reduced pressure using an evaporator with bath at 35–45 °C.

*(2R)-N-{(E)-3-[((2E)-3,7-Dimethylocta-2,6-dien-1-yl)oxy]acryloyl}bornane-10,2-sultam* (**6**). The following reaction was carried out under Ar. To a cooled (0 °C) stirred solution of **5** (302 mg, 1.13 mmol) in CH_2_Cl_2_ (11 mL) were added geraniol (218 μL, 1.24 mmol) and *n*-Bu_3_P (42 μL, 0.17 mmol). The mixture was stirred at 0 °C for 30 min, diluted with H_2_O (20 mL), and extracted with CH_2_Cl_2_ (10 mL × 3). The combined extracts were washed with saturated brine (20 mL), dried and concentrated under reduced pressure. The residue was purified by column chromatography on silica gel (EtOAc/hexane, 1:30) to provide 368 mg (77%) of **6** as a colorless oil: TLC *R_f_* 0.54 (EtOAc/hexane, 1:3); [α]_D_^19^–59.2 (*c* 1.19, CHCl_3_); IR (neat) 2962, 2885, 1678, 1608 cm^−1^; ^1^H-NMR (500 MHz): δ 0.97 (s, 3H), 1.18 (s, 3H), 1.34–1.45 (m, 2H), 1.60 (br s, 3H), 1.68 (br s, 3H), 1.71 (br s, 3H), 1.86–1.91 (m, 3H), 2.05–2.17 (m, 6H), 3.43 (d, 1H, *J* = 13.8 Hz), 3.48 (d, 1H, *J* = 13.8 Hz), 3.91 (dd, 1H, *J* = 4.9, 7.7 Hz), 4.45 (d, 2H, *J* = 6.9 Hz), 5.08 (m, 1H), 5.37 (qt, 1H, *J* = 1.0, 6.9 Hz), 5.97 (d, 1H, *J* = 12.1 Hz), 7.70 (d, 1H, *J* = 12.1 Hz); ^13^C-NMR (125 MHz) δ16.6, 17.6, 19.9, 20.7, 25.6, 26.1, 26.5, 32.7, 38.5, 39.4, 44.6, 47.7, 48.2, 53.0, 65.0, 68.1, 97.0, 117.5, 123.5, 131.9, 143.4, 163.3, 164.9; HRMS calcd for C_23_H_35_NO_4_S (M^+^) *m/z* 421.2287, found 421.2286.

*(2R)-N-[(2R,3S)-2-Formyl-3,7-dimethyl-3-vinyloct-6-enoyl]bornane-10,2-sultam* (**7a**) and *(2R)-N-[(2S,3R)]-isomer* (**7b**). A solution of **6** (400 mg, 949 μmol) and BHT (10.5 mg, 47.5 μmol) in toluene (50 mL) was stirred at 140 °C for 65 h in a sealed tube and concentrated under reduced pressure. The residue was purified by column chromatography on silica gel (EtOAc/hexane, 1:30) to provide 289 mg (72%) of **7a** and 30.9 mg (8%) of **7b**. Compound **7a** was obtained as white crystals: mp 84–87 °C; TLC *R_f_* 0.49 (EtOAc/hexane, 1:3); [α]_D_^21^–77.9 (*c* 2.55, CHCl_3_); IR (neat) 2960, 2925, 1730, 1680 cm^−1^; ^1^H-NMR (500 MHz) δ 0.98 (s, 3H), 1.15 (s, 3H), 1.26 (s, 3H), 1.37–1.46 (m, 3H), 1.56 (br s, 3H), 1.65 (br s, 3H), 1.67 (m, 1H), 1.87–1.96 (m, 5H), 2.06-2.15 (m, 2H), 3.44 (d, 1H, *J* = 13.8 Hz), 3.51 (d, 1H, *J* = 13.8 Hz), 3.96 (dd, 1H, *J* = 5.2, 7.4 Hz), 4.01 (d, 1H, *J* = 2.3 Hz), 5.02 (m, 1H), 5.07 (d, 1H, *J* = 17.4 Hz), 5.21 (d, 1H, *J* = 10.6 Hz), 5.92 (dd, 1H, *J* = 10.6, 17.4 Hz), 9.61 (d, 1H, *J* = 2.3 Hz); ^13^C-NMR (125 MHz) δ 17.6, 19.7, 19.9, 20.7, 22.2, 25.6, 26.4, 32.9, 38.5 (2C), 44.8, 45.5, 47.7, 48.1, 53.2, 65.4 (2C), 115.2, 123.7, 131.9, 142.3, 167.5, 197.3; HRMS calcd for C_23_H_35_NO_4_S (M^+^) *m/z* 421.2287, found 421.2283. Compound **7b** was obtained as white crystals: mp 81–87 °C; TLC *R_f_* 0.61 (EtOAc/hexane, 1:3); [α]_D_^17^+38.5 (*c* 0.965, CHCl_3_); IR (neat) 2960, 2925, 1730, 1700 cm^−1^; ^1^H-NMR (500 MHz) δ 0.95 (s, 3H), 1.10 (s, 3H), 1.31 (s, 3H), 1.34–1.43 (m, 2H), 1.54-1.68 (m, 2H), 1.56 (br s, 3H), 1.65 (br s, 3H), 1.88-1.93 (m, 5H), 2.08 (dd, 1H, *J* = 7.8, 13.9 Hz), 2.28 (m, 1H), 3.43 (d, 1H, *J* = 13.7 Hz), 3.48 (d, 1H, *J* = 13.7 Hz), 3.90 (dd, 1H, *J* = 4.9, 7.8 Hz), 4.21 (d, 1H, *J* = 0.9 Hz), 5.04 (m, 1H), 5.12 (dd, 1H, *J* = 0.6, 17.5 Hz), 5.26 (dd, 1H, *J* = 0.6, 10.8 Hz), 6.01 (dd, 1H, *J* = 10.8, 17.5 Hz), 9.60 (d, 1H, *J* = 0.9 Hz); ^13^C-NMR (125 MHz) δ 17.6, 19.3, 19.9, 20.4, 22.2, 25.7, 26.5, 32.7, 38.2, 38.9, 42.9, 44.5, 47.8, 48.2, 53.1, 65.1, 65.3, 115.1, 124.0, 131.7, 143.5, 166.3, 197.7; HRMS calcd for C_23_H_35_NO_4_S (M^+^) *m/z* 421.2287, found 421.2281.

*(2R)-N-{(E)-3-[((2Z)-3,7-Dimethylocta-2,6-dien-1-yl)oxy]acryloyl}bornane-10,2-sultam* (**8**). As described for the preparation of **6**, compound **5** (210 mg, 785 μmol) and nerol (155 μL, 882 μmol) were treated with *n*-Bu_3_P (32 μL, 0.12 mmol) in CH_2_Cl_2_ (8 mL) to provide 234 mg (71%) of **8** as white crystals: mp 62–64 °C; TLC *R_f_* 0.52 (EtOAc/hexane, 1:3); [α]_D_^26^–71.0 (*c* 1.22, CHCl_3_); IR (neat) 2964, 2884, 1677, 1607 cm^−1^; ^1^H-NMR (500 MHz) δ 0.97 (s, 3H), 1.18 (s, 3H), 1.34–1.45 (m, 2H), 1.60 (br s, 3H), 1.69 (br s, 3H), 1.78 (br s, 3H), 1.87-1.91 (m, 3H), 2.05–2.17 (m, 6H), 3.43 (d, 1H, *J* = 13.7 Hz), 3.48 (d, 1H, *J* = 13.7 Hz), 3.91 (dd, 1H, *J* = 4.9, 7.8 Hz), 4.41 (d, 2H, *J* = 7.0 Hz), 5.08 (m, 1H), 5.39 (t, 1H, *J* = 7.0 Hz), 5.96 (d, 1H, *J* = 12.0 Hz), 7.69 (d, 1H, *J* = 12.0 Hz); ^13^C-NMR (125 MHz) δ 17.6, 19.9, 20.8, 23.5, 25.7, 26.5 (2C), 32.3, 32.8, 38.6, 44.7, 47.8, 48.2, 53.1, 65.0, 67.9, 97.0, 118.5, 123.3, 132.5, 143.8, 163.4, 165.0; HRMS calcd for C_23_H_35_NO_4_S (M^+^) *m/z* 421.2287, found 421.2287.

*(2R)-N-[(2R,3R)-2-Formyl-3,7-dimethyl-3-vinyloct-6-enoyl]bornane-10,2-sultam* (**7c**) and *(2R)-N-[(2S,3S)]-isomer* (**7d**). As described for the preparation of **7a** and **7b** from **6**, a solution of **8** (223 mg, 529 μmol) and BHT (5.8 mg, 26 μmol) in toluene (27 mL) was heated at 140 °C for 26 h to provide 147 mg (66%) of a mixture of **7c** and **7a** (**7c**/**7a** = 19:1) and 25.0 mg (11%) of a mixture of **7d** and **7b** (**7d**/**7b** = 10:1), and 27.9 mg (13%) of **8** was recovered. A mixture of **7c** and **7a** (**7c**/**7a** = 19:1) was obtained as a colorless oil: TLC *R_f_* 0.49 (EtOAc/hexane, 1:3); [α]_D_^28^–82.4 (*c* 1.26, CHCl_3_); IR (neat) 2965, 2930, 1727, 1684 cm^−1^; ^1^H-NMR (500 MHz) for **7c** δ 0.97 (s, 3H), 1.16 (s, 3H), 1.26 (s, 3H), 1.34–1.49 (m, 3H), 1.55 (br s, 3H), 1.65 (br s, 3H), 1.68 (m, 1H), 1.84–1.93 (m, 5H), 2.03–2.09 (m, 2H), 3.43 (d, 1H, *J* = 13.8 Hz), 3.50 (d, 1H, *J* = 13.8 Hz), 3.89 (d, 1H, *J* = 3.5 Hz), 3.92 (dd, 1H, *J* = 5.5, 7.4 Hz), 5.02 (m, 1H), 5.02 (dd, 1H, *J* = 1.0, 17.4 Hz), 5.14 (dd, 1H, *J* = 1.0, 10.9 Hz), 6.02 (dd, 1H, *J* = 10.9, 17.4 Hz), 9.66 (d, 1H, *J* = 3.5 Hz); ^13^C-NMR (125 MHz) for **7c** δ 17.6, 18.9, 19.9, 20.7, 22.2, 25.6, 26.4, 33.0, 38.2, 39.5, 44.7, 45.8, 47.7, 48.1, 53.3, 65.4, 65.5, 115.2, 123.7, 131.8, 141.7, 167.9, 197.8; HRMS calcd for C_23_H_35_NO_4_S (M^+^) *m/z* 421.2287, found 421.2289. A mixture of **7d** and **7b** (**7d**/**7b** = 10:1) was obtained as a colorless oil: TLC *R_f_* 0.61 (EtOAc/hexane, 1:3); [α]_D_^26^+2.9 (*c* 1.25, CHCl_3_); IR (neat) 2964, 2924, 1728, 1697 cm^−1^; ^1^H-NMR (500 MHz) for **7d** δ 0.95 (s, 3H), 1.09 (s, 3H), 1.31–1.42 (m, 2H), 1.34 (s, 3H), 1.57 (m, 1H), 1.57 (br s, 3H), 1.65 (br s, 3H), 1.76 (m, 1H), 1.87–1.95 (m, 5H), 2.07 (dd, 1H, *J* = 7.9, 14.0 Hz), 2.25 (m, 1H), 3.43 (d, 1H, *J* = 13.9 Hz), 3.49 (d, 1H, *J* = 13.9 Hz), 3.90 (dd, 1H, *J* = 4.9, 7.9 Hz), 4.19 (d, 1H, *J* = 1.1 Hz), 5.06 (m, 1H), 5.06 (d, 1H, *J* = 17.2 Hz), 5.17 (d, 1H, *J* = 10.9 Hz), 6.14 (dd, 1H, *J* = 10.9, 17.2 Hz), 9.68 (d, 1H, *J* = 1.1 Hz); ^13^C-NMR (125 MHz) for **7d** δ 17.6, 19.9, 20.4, 21.2, 23.4, 25.6, 26.4, 32.7, 38.2, 39.1, 43.3, 44.5, 47.7, 48.2, 53.1, 65.3, 66.2, 114.7, 123.9, 131.7, 142.6, 166.3, 197.0; HRMS calcd for C_23_H_35_NO_4_S (M^+^) *m/z* 421.2287, found 421.2288.

*Epimerization of* **7a**. To a stirred solution of **7a** (7.9 mg, 19 μmol) in CH_2_Cl_2_ (1 mL) was added DBU (3.6 μL, 24 μmol). The mixture was stirred at room temperature for 45 min, diluted with 1 M aqueous HCl (1 mL), and extracted with CH_2_Cl_2_ (2 mL × 3). The combined extracts were washed with saturated brine (1 mL), dried and concentrated under reduced pressure. The residue was purified by column chromatography on silica gel (EtOAc/hexane, 1:25) to provide 1.1 mg (14%) of **7d** and 6.7 mg (85%) of **7a** was recovered.

*(1RS,3R)-1-(4-Methoxyphenyl)-3,7-dimethyl-3-vinyloct-6-enol* (**10**). To a cooled (0 °C) stirred solution of **7a** (233 mg, 553 μmol) in THF/H_2_O (1:1, 5 mL) was added 1.00 M aqueous KOH (1.11 mL, 1.11 mmol). The mixture was stirred at room temperature for 24 h, quenched with saturated aqueous NH_4_Cl (2 mL), diluted with H_2_O (2 mL), and extracted with Et_2_O (5 mL × 3). The combined extracts were washed with saturated brine (15 mL) and dried to provide a solution of aldehyde **9** in Et_2_O, which was used in the next step without further evaporation and purification.

The following reaction was carried out under Ar. To a cooled (0 °C) stirred solution of aldehyde **9** in Et_2_O obtained above was added 4-methoxyphenylmagnesium bromide (1.50 M solution in Et_2_O, total 6.27 mL, total 9.41 mmol) in ten times over a period of 2 h. The mixture was quenched with saturated aqueous NH_4_Cl (30 mL), diluted H_2_O (10 mL), and extracted with CH_2_Cl_2_ (40 mL × 3). The combined extracts were dried and concentrated under reduced pressure. The residue was purified by column chromatography on silica gel (EtOAc/hexane, 1:40) to provide 86.7 mg (55%) of **10** and 114 mg (96%) of camphorsultam. Compound **10** (dr = 1:1) was obtained as a colorless oil: TLC *R_f_* 0.61 (EtOAc/hexane, 1:3); IR (neat) 3442, 2965, 2924, 1612, 1512 cm^−1^; ^1^H-NMR (500 MHz) δ 1.10 (s, 3H × 1/2), 1.11 (s, 3H × 1/2), 1.34 (t, 2H × 1/2, *J* = 8.5 Hz), 1.40–1.43 (m, 2H × 1/2), 1.57 (br s, 3H × 1/2), 1.59 (br s, 3H × 1/2), 1.66 (br s, 3H × 1/2), 1.67 (br s, 3H × 1/2), 1.80–1.93 (m, 4H), 3.79 (s, 3H), 4.74 (dd, 1H × 1/2, *J* = 2.6, 8.6 Hz), 4.79 (dd, 1H × 1/2, *J* = 2.6, 9.3 Hz), 5.02 (dd, 1H × 1/2, *J* = 1.1, 17.7 Hz), 5.07 (dd, 1H × 1/2, *J* = 1.1, 10.8 Hz), 5.07 (m, 1H), 5.07 (dd, 1H × 1/2, *J* = 0.9, 17.7 Hz), 5.14 (dd, 1H × 1/2, *J* = 0.9, 10.8 Hz), 5.83 (dd, 1H × 1/2, *J* = 10.8, 17.7 Hz), 5.97 (dd, 1H × 1/2, *J* = 10.8, 17.7 Hz), 6.86 (d, 2H × 1/2, *J* = 8.8 Hz), 6.86 (d, 2H × 1/2, *J* = 8.6 Hz), 7.24 (d, 2H × 1/2, *J* = 8.8 Hz), 7.25 (d, 2H × 1/2, *J* = 8.6 Hz); ^13^C-NMR (125 MHz) δ 17.6, 21.3 (1/2C), 22.5 (1/2C), 22.7 (1/2C), 23.5 (1/2C), 25.7, 39.5, 40.5 (1/2C), 42.5 (1/2C), 50.3 (1/2C), 51.1 (1/2C), 55.3, 71.4 (1/2C), 71.5 (1/2C), 112.2 (1/2C), 112.9 (1/2C), 113.7, 113.8, 124.6 (1/2C), 124.8 (1/2C), 126.9 (2C), 131.2 (1/2C), 131.3 (1/2C), 137.7 (1/2C), 138.3 (1/2C), 147.4 (1/2C), 147.7 (1/2C), 158.8; HRMS calcd for C_19_H_28_O_2_ (M^+^) *m/z* 288.2089, found 288.2090.

*(1E,3S)-1-(4-Methoxyphenyl)-3,7-dimethyl-3-vinylocta-1,6-diene* (**11**). The following reaction was carried out under Ar. To a stirred solution of **10** (22.5 mg, 78.0 μmol) in pyridine (1 mL) was added POCl_3_ (8.6 μL, 95 μmol). The mixture was refluxed for 4 h, diluted with EtOAc (15 mL), and washed with H_2_O (10 mL) and saturated brine (10 mL). The organic layer was dried and concentrated under reduced pressure. The residue was purified by column chromatography on silica gel (EtOAc/hexane, 1:100) to provide 18.9 mg (90%) of **11** as a colorless oil: TLC *R_f_* 0.80 (EtOAc/hexane, 1:3); [α]_D_^25^+28.4 (*c* 0.855, CHCl_3_); IR (neat) 2966, 2916, 1609, 1511 cm^−1^; ^1^H-NMR (500 MHz) δ 1.20 (s, 3H), 1.48–1.51 (m, 2H), 1.58 (br s, 3H), 1.67 (br s, 3H), 1.93–1.98 (m, 2H), 3.80 (s, 3H), 5.01 (dd, 1H, *J* = 1.4, 17.5 Hz), 5.03 (dd, 1H, *J* = 1.4, 10.7 Hz), 5.11 (m, 1H), 5.88 (dd, 1H, *J* = 10.7, 17.5 Hz), 6.06 (d, 1H, *J* = 16.4 Hz), 6.26 (d, 1H, *J* = 16.4 Hz), 6.83 (d, 2H, *J* = 8.7 Hz), 7.29 (d, 2H, *J* = 8.7 Hz); ^13^C-NMR (125 MHz) δ 17.6, 23.2, 23.4, 25.7, 41.3, 42.5, 55.3, 111.8, 113.9 (2C), 124.8, 126.5, 127.1 (2C), 130.7, 131.3, 135.8, 146.0, 158.7; HRMS calcd for C_19_H_26_O (M^+^) *m/z* 270.1984, found 270.1983.

*(+)-Bakuchiol* (**4**). The following reaction was carried out under Ar. To a cooled (0 °C) solution of **11** (30.2 mg, 112 μmol) in Et_2_O (1 mL) was added MeMgI (0.500 M solution in Et_2_O, 1.57 mL, 785 μmol). The solvent was removed under reduced pressure. The residue was heated at 180 °C for 15 min and cooled to room temperature. The mixture was quenched with 1 M aqueous HCl (2 mL), diluted with H_2_O (2 mL), and extracted with CH_2_Cl_2_ (5 mL × 3). The combined extracts were dried and concentrated under reduced pressure. The residue was purified by column chromatography on silica gel (EtOAc/hexane, 1:60) to provide 26.1 mg (91%) of **4** as a pale yellow oil: TLC *R_f_* 0.63 (EtOAc/hexane, 1:3); [α]_D_^29^ + 25.6 (*c* 0.795, CHCl_3_); IR (neat) 3359, 2967, 2924, 1610, 1513 cm^−1^; ^1^H-NMR (500 MHz) δ 1.19 (s, 3H), 1.47-1.51 (m, 2H), 1.58 (br s, 3H), 1.67 (br s, 3H), 1.93–1.97 (m, 2H), 4.85 (br, 1H, OH), 5.01 (dd, 1H, *J* = 1.5, 17.4 Hz), 5.03 (dd, 1H, *J* = 1.5, 10.8 Hz), 5.11 (m, 1H), 5.88 (dd, 1H, *J* = 10.8, 17.4 Hz), 6.05 (d, 1H, *J* = 16.2 Hz), 6.25 (d, 1H, *J* = 16.2 Hz), 6.76 (d, 2H, *J* = 8.6 Hz), 7.24 (d, 2H, *J* = 8.6 Hz); ^13^C-NMR (125 MHz) δ 17.6, 23.2, 23.3, 25.7, 41.3, 42.5, 111.9, 115.3 (2C), 124.8, 126.4, 127.4 (2C), 130.9, 131.3, 135.9, 145.9, 154.6; HRMS calcd for C_18_H_24_O (M^+^) *m/z* 256.1827, found 256.1829.

*(2R)-N-[(1R,2S,5R,6R)-6-Hydroxy-5-isopropenyl-2-methyl-2-vinylcyclohexanecarbonyl]bornane-10,2-sultam* (**12aa**) and its diastereomers. A solution of **7a** (22.8 mg, 54.1 μmol) and BHT (a crystal) in toluene (6 mL) was stirred at 160 °C for 50 h in a sealed tube and concentrated under reduced pressure. The residue was purified by column chromatography on silica gel (EtOAc/hexane, 1:15) to provide 4.9 mg (21%) of **12aa**, 3.2 mg (14%) of **12ab**, 3.3 mg (14%) of **12ac**, and 1.7 mg (7%) of **12ad**. Compound **12aa** was obtained as white crystals: mp 198–200 °C; TLC *R_f_* 0.43 (EtOAc/hexane, 1:2); [α]_D_^21^–10.5 (*c* 0.27, CHCl_3_); IR (neat) 3520, 2960, 1695 cm^−1^; ^1^H-NMR (500 MHz) δ 0.97 (s, 3H), 1.16 (s, 3H), 1.17 (s, 3H), 1.36–1.44 (m, 2H), 1.51–1.56 (m, 2H), 1.67-1.79 (m, 2H), 1.73 (br s, 3H), 1.86–1.92 (m, 3H), 2.02 (d, 1H, *J* = 8.3 Hz, OH), 2.08–2.15 (m, 3H), 2.99 (d, 1H, *J* = 10.6 Hz), 3.47 (d, 1H, *J* = 13.9 Hz), 3.53 (d, 1H, *J* = 13.9 Hz), 3.94 (dt, 1H, *J* = 8.3, 10.6 Hz), 4.00 (dd, 1H, *J* = 5.1, 7.7 Hz), 4.83 (s, 1H), 4.85 (s, 1H), 4.98 (dd, 1H, *J* = 1.2, 17.5 Hz), 5.12 (dd, 1H, *J* = 1.2, 11.2 Hz), 6.43 (dd, 1H, *J* = 11.2, 17.5 Hz); ^13^C-NMR (125 MHz) δ 19.2, 20.0, 20.9, 26.4, 26.5, 27.8, 33.0, 38.7, 39.0, 42.5, 44.8, 47.6, 47.7, 53.6, 54.1, 60.7, 65.9, 70.3, 112.5, 113.1, 141.5, 146.2, 171.9; HRMS calcd for C_23_H_35_NO_4_S (M^+^) *m/z* 421.2287, found 421.2291.

*(2R)-N-[(1R,2R,5R,6R)-6-Hydroxy-5-isopropenyl-2-methyl-2-vinylcyclohexanecarbonyl]bornane-10,2-sultam* (**12ca**) and its diastereomers. As described for the preparation of **12aa** and its diastereomers from **7a**, a solution of **7c** (23.5 mg, 55.7 μmol) and BHT (a crystal) in toluene (6 mL) was heated at 160 °C for 40 h to provide 8.7 mg (37%) of **12ca**, 9.2 mg (39%) of a mixture of **12cb** and **12cc**, and 0.9 mg (4%) of **12cd**. Compound **12ca** was obtained as white crystals: TLC *R_f_* 0.32 (EtOAc/hexane, 1:2); ^1^H-NMR (500 MHz) δ 0.95 (s, 3H), 1.15 (s, 3H), 1.19 (s, 3H), 1.23–1.41 (m, 3H), 1.50–1.70 (m, 3H), 1.78 (br s, 3H), 1.81–1.96 (m, 3H), 2.02–2.06 (m, 3H), 2.10 (dt, 1H, *J* = 5.0, 10.6 Hz), 3.00 (d, 1H, *J* = 10.5 Hz), 3.45 (d, 1H, *J* = 13.9 Hz), 3.50 (d, 1H, *J* = 13.9 Hz), 3.95 (dd, 1H, *J* = 5.2, 7.8 Hz), 4.00 (q, 1H, *J* = 10.5 Hz), 4.85 (s, 1H), 4.86 (s, 1H), 4.90 (d, 1H, *J* = 10.6 Hz), 4.98 (d, 1H, *J* = 17.5 Hz), 6.00 (dd, 1H, *J* = 10.6, 17.5 Hz).

*(2R)-N-{(E)-3-[((2E)-6-(tert-Butyldiphenysilyloxy)-3-methylhex-2-en-1-yl)oxy]acryloyl}bornane-10,2-sultam* (**14**). As described for the preparation of **6**, compound **5** (109 mg, 408 μmol) and **13** (165 mg, 448 μmol) were treated with *n*-Bu_3_P (15 μL, 61 μmol) in CH_2_Cl_2_ (4 mL) to provide 223 mg (86%) of **14** as white crystals: mp 74–77 °C; TLC *R_f_* 0.78 (EtOAc/toluene, 1:4); [α]_D_^26^ – 45.6 (*c* 1.02, CHCl_3_); IR (neat) 2958, 2858, 1678, 1608 cm^−1^; ^1^H-NMR (500 MHz) δ 0.97 (s, 3H), 1.05 (s, 9H), 1.17 (s, 3H), 1.36–1.45 (m, 2H), 1.65–1.70 (m, 2H), 1.67 (s, 3H), 1.86–1.91 (m, 3H), 2.08 (dd, 1H, *J* = 7.8, 13.8 Hz), 2.13 (t, 2H, *J* = 7.8 Hz), 2.15 (m, 1H), 3.42 (d, 1H, *J* = 13.8 Hz), 3.48 (d, 1H, *J* = 13.8 Hz), 3.64 (t, 2H, *J* = 6.3 Hz), 3.91 (dd, 1H, *J* = 4.9, 7.8 Hz), 4.41 (d, 2H, *J* = 7.1 Hz), 5.35 (t, 1H, *J* = 7.1 Hz), 5.96 (d, 1H, *J* = 12.0 Hz), 7.36–7.44 (m, 6H), 7.65–7.67 (m, 4H), 7.70 (d, 1H, *J* = 12.0 Hz); ^13^C-NMR (125 MHz) δ 16.6, 19.2, 19.9, 20.8, 26.5, 26.9 (3C), 30.5, 32.8, 35.7, 38.6, 44.7, 47.8, 48.2, 53.1, 63.3, 65.0, 68.1, 97.0, 117.6, 127.6 (4C), 129.5 (2C), 134.0 (2C), 135.6 (4C), 143.4, 163.4, 165.0; HRMS calcd for C_32_H_40_NO_5_SSi (M^+^–*t*-C_4_H_9_) *m/z* 578.2396, found 578.2398.

*(2R)-N-[(2R,3S)-6-(tert-Butyldiphenysilyloxy)-2-formyl-3-methyl-3-vinylhexanoyl]bornane-10,2-sultam* (**15a**) and *(2R)-N-[(2S,3R)]-Isomer* (**15b**). As described for the preparation of **7a** and **7b** from **6**, a solution of **14** (209 mg, 329 μmol) and BHT (3.6 mg, 16 μmol) in toluene (17 mL) was heated at 140 °C for 71 h to provide 150 mg (72%) of **15a** and 32.1 mg (15%) of **15b**. Compound **15a** was obtained as a colorless oil: TLC *R_f_* 0.59 (EtOAc/toluene, 1:5); [α]_D_^23^–88.2 (*c* 1.46, CHCl_3_); IR (neat) 2961, 2859, 1731, 1686 cm^−1^; ^1^H-NMR (500 MHz) δ 0.95 (s, 3H), 1.14 (s, 9H), 1.11 (s, 3H), 1.23 (s, 3H), 1.35–1.40 (m, 2H), 1.48-1.54 (m, 2H), 1.74 (m, 1H), 1.83–1.91 (m, 4H), 2.07–2.13 (m, 2H), 3.43 (d, 1H, *J* = 13.8 Hz), 3.50 (d, 1H, *J* = 13.8 Hz), 3.60 (t, 2H, *J* = 6.3 Hz), 3.95 (dd, 1H, *J* = 5.4, 7.5 Hz), 4.01 (d, 1H, *J* = 2.5 Hz), 5.05 (d, 1H, *J* = 17.5 Hz), 5.19 (d, 1H, *J* = 10.9 Hz), 5.88 (dd, 1H, *J* = 10.9, 17.5 Hz), 7.35–7.43 (m, 6H), 7.63–7.65 (m, 4H), 9.60 (d, 1H, *J* = 2.5 Hz); ^13^C-NMR (125 MHz) δ 19.2, 19.8, 19.9, 20.8, 26.4, 26.7, 26.8 (3C), 32.9, 34.5, 38.5, 44.7, 45.3, 47.7, 48.1, 53.2, 63.9, 65.4, 65.5, 115.3, 127.6 (4C), 129.5 (2C), 134.0 (2C), 135.6 (4C), 142.3, 167.4, 197.2; HRMS calcd for C_32_H_40_NO_5_SSi (M^+^–*t*-C_4_H_9_) *m/z* 578.2396, found 578.2401. Compound **15b** was obtained as a colorless oil: TLC *R_f_* 0.69 (EtOAc/toluene, 1:5); [α]_D_^24^+6.7 (*c* 1.50, CHCl_3_); IR (neat) 2961, 2859, 1728, 1696 cm^−1^; ^1^H-NMR (500 MHz) δ 0.94 (s, 3H), 1.04 (s, 9H), 1.10 (s, 3H), 1.27 (s, 3H), 1.33–1.39 (m, 2H), 1.51 (m, 1H), 1.62 (m, 1H), 1.70 (m, 1H), 1.87–1.91 (m, 4H), 2.06 (dd, 1H, *J* = 7.8, 14.0 Hz), 2.26 (m, 1H), 3.41 (d, 1H, *J* = 13.7 Hz), 3.48 (d, 1H, *J* = 13.7 Hz), 3.61 (t, 2H, *J* = 6.5 Hz), 3.87 (dd, 1H, *J* = 4.9, 7.8 Hz), 4.20 (s, 1H), 5.09 (d, 1H, *J* = 17.5 Hz), 5.23 (d, 1H, *J* = 10.7 Hz), 5.96 (dd, 1H, *J* = 10.7, 17.5 Hz), 7.36–7.43 (m, 6H), 7.64–7.66 (m, 4H), 9.59 (s, 1H); ^13^C-NMR (125 MHz) δ 19.2, 19.5, 19.9, 20.4, 26.4, 26.7, 26.9 (3C), 32.8, 34.9, 38.2, 42.7, 44.5, 47.7, 48.2, 53.1, 64.0, 65.2, 65.3, 115.1, 127.6 (4C), 129.5 (2C), 134.0 (2C), 135.6 (4C), 143.4, 166.3, 197.7; HRMS calcd for C_32_H_40_NO_5_SSi (M^+^–*t*-C_4_H_9_) *m/z* 578.2396, found 578.2389.

*(2R)-N-{(E)-3-[((2E)-5-(1,3-Dioxolan-2-yl)-3-methylpent-2-en-1-yl)oxy]acryloyl}bornane-10,2-sultam* (**17**). As described for the preparation of **6**, compound **5** (171 mg, 640 μmol) and **16** (121 mg, 703 μmol) were treated with *n*-Bu_3_P (24 μL, 97 μmol) in CH_2_Cl_2_ (6 mL) to provide 222 mg (79%) of **17** as a colorless oil: TLC *R_f_* 0.67 (EtOAc/toluene, 1:3); [α]_D_^25^–59.7 (*c* 1.16, CHCl_3_); IR (neat) 2958, 2885, 1677, 1609 cm^−1^; ^1^H-NMR (500 MHz) δ 0.97 (s, 3H), 1.18 (s, 3H), 1.34–1.45 (m, 2H), 1.72 (s, 3H), 1.77–1.81 (m, 2H), 1.87–1.91 (m, 3H), 2.07 (dd, 1H, *J* = 7.8, 13.9 Hz), 2.14 (m, 1H), 2.18 (t, 2H, *J* = 8.1 Hz), 3.43 (d, 1H, *J* = 13.8 Hz), 3.48 (d, 1H, *J* = 13.8 Hz), 3.84–3.86 (m, 2H), 3.91 (dd, 1H, *J* = 5.0, 7.8 Hz), 3.95–3.98 (m, 2H), 4.45 (d, 2H, *J* = 6.9 Hz), 4.86 (t, 1H, *J* = 4.7 Hz), 5.41 (t, 1H, *J* = 6.9 Hz), 5.96 (d, 1H, *J* = 12.1 Hz), 7.69 (d, 1H, *J* = 12.1 Hz); ^13^C-NMR (125 MHz) δ 16.7, 19.9, 20.8, 26.5, 31.8, 32.7, 33.6, 38.5, 44.6, 47.7, 48.2, 53.0, 64.9 (2C), 65.0, 68.0, 97.0, 104.0, 117.8, 142.7, 163.3, 164.9; HRMS calcd for C_22_H_33_NO_6_S (M^+^) *m/z* 439.2029, found 439.2035.

*(2R)-N-[(2R,3S)-5-(1,3-Dioxolan-2-yl)-2-formyl-3-methyl-3-vinylpentanoyl]bornane-10,2-sultam* (**18a**) and *(2R)-N-[(2S,3R)]-Isomer* (**18b**). As described for the preparation of **7a** and **7b** from **6**, a solution of **17** (219 mg, 498 μmol) and BHT (5.5 mg, 25 μmol) in toluene (25 mL) was heated at 140 °C for 116 h to provide 159 mg (73%) of **18a** and 34.0 mg (16%) of **18b**. Compound **18a** was obtained as white crystals: mp 116–118 °C; TLC *R_f_* 0.67 (EtOAc/toluene, 1:2); [α]_D_^21^–119 (*c* 1.34, CHCl_3_); IR (neat) 2964, 2886, 1731, 1684 cm^−1^; ^1^H-NMR (500 MHz) δ 0.98 (s, 3H), 1.16 (s, 3H), 1.24 (s, 3H), 1.34–1.43 (m, 2H), 1.53–1.63 (m, 2H), 1.81 (m, 1H), 1.88–1.96 (m, 4H), 2.11–2.12 (m, 2H), 3.44 (d, 1H, *J* = 13.7 Hz), 3.51 (d, 1H, *J* = 13.7 Hz), 3.80–3.83 (m, 2H), 3.91–3.94 (m, 2H), 3.96 (t, 1H, *J* = 6.6 Hz), 4.01 (d, 1H, *J* = 2.3 Hz), 4.81 (t, 1H, *J* = 4.2 Hz), 5.08 (d, 1H, *J* = 17.5 Hz), 5.22 (d, 1H, *J* = 10.6 Hz), 5.89 (dd, 1H, *J* = 10.6, 17.5 Hz), 9.62 (d, 1H, *J* = 2.3 Hz); ^13^C-NMR (125 MHz) δ 19.7, 19.9, 20.8, 26.4, 28.0, 32.0, 33.0, 38.5, 44.8, 45.0, 47.7, 48.1, 53.2, 64.9 (2C), 65.4 (2C), 104.3, 115.6, 141.9, 167.4, 197.1; HRMS calcd for C_22_H_33_NO_6_S (M^+^) *m/z* 439.2029, found 439.2036. Compound **18b** was obtained as a colorless oil: TLC *R_f_* 0.75 (EtOAc/toluene, 1:2); [α]_D_^22^+10.4 (*c* 1.67, CHCl_3_); IR (neat) 2962, 2885, 1728, 1697 cm^−1^; ^1^H-NMR (500 MHz) δ 0.94 (s, 3H), 1.10 (s, 3H), 1.29 (s, 3H), 1.32–1.42 (m, 2H), 1.58–1.65 (m, 2H), 1.74 (m, 1H), 1.87–1.94 (m, 4H), 2.07 (dd, 1H, *J* = 7.8, 13.8 Hz), 2.26 (m, 1H), 3.43 (d, 1H, *J* = 13.9 Hz), 3.48 (d, 1H, *J* = 13.9 Hz), 3.81–3.85 (m, 2H), 3.90–3.95 (m, 3H), 4.21 (s, 1H), 4.82 (t, 1H, *J* = 4.4 Hz), 5.12 (d, 1H, *J* = 17.5 Hz), 5.26 (d, 1H, *J* = 10.9 Hz), 5.98 (dd, 1H, *J* = 10.9, 17.5 Hz), 9.61 (s, 1H); ^13^C-NMR (125 MHz) δ 19.2, 19.9, 20.4, 26.4, 28.1, 32.6, 32.7, 38.1, 42.5, 44.5, 47.7, 48.2, 53.0, 64.9 (2C), 65.2 (2C), 104.4, 115.5, 143.0, 166.2, 197.5; HRMS calcd for C_22_H_33_NO_6_S (M^+^) *m/z* 439.2029, found 439.2032.

*(2R)-N-[(2R,3S)-2-(Acetoxymethyl)-3,7-dimethyl-3-vinyloct-6-enoyl]bornane-10,2-sultam* (**19a**). To a cooled (0 °C) stirred solution of **7a** (158 mg, 375 μmol) in EtOH (4 mL) was added NaBH_4_ (14.2 mg, 375 μmol). The mixture was stirred at 0 °C for 4 h, quenched with saturated aqueous NH_4_Cl (1 mL), diluted with H_2_O (20 mL), and extracted with CH_2_Cl_2_ (10 mL × 3). The combined extracts were dried and concentrated under reduced pressure to provide crude alcohol (152 mg), which was used in the next step without further purification. 

To a cooled (0 °C) stirred solution of crude alcohol in CH_2_Cl_2_ (4 mL) were added Ac_2_O (85 μL, 0.90 mmol), Et_3_N (150 μL, 1.08 mmol), and DMAP (4.4 mg, 36 μmol). The mixture was stirred at room temperature for 2.5 h, diluted with CH_2_Cl_2_ (20 mL), and washed with H_2_O (10 mL × 2). The combined aqueous layers were extracted with CH_2_Cl_2_ (30 mL). The combined organic layer and extract were dried and concentrated under reduced pressure. The residue was purified by column chromatography on silica gel (EtOAc/hexane, 1:15) to provide 137 mg (78% for 2 steps) of **19a** as a colorless oil: TLC *R_f_* 0.61 (EtOAc/hexane, 1:2); [α]_D_^21^–38.4 (*c* 1.46, CHCl_3_); IR (neat) 2964, 2884, 1745, 1690 cm^−1^; ^1^H-NMR (500 MHz) δ 0.96 (s, 3H), 1.10 (s, 3H), 1.13 (s, 3H), 1.27–1.47 (m, 3H), 1.55 (br s, 3H), 1.64 (br s, 3H), 1.65 (m, 1H), 1.81–1.92 (m, 5H), 1.99 (s, 3H), 2.09-2.13 (m, 2H), 3.35 (m, 1H), 3.43 (d, 1H, *J* = 13.7 Hz), 3.50 (d, 1H, *J* = 13.7 Hz), 3.94 (t, 1H, *J* = 6.4 Hz), 4.22 (t, 1H, *J* = 10.6 Hz), 4.37 (dd, 1H, *J* = 3.4, 10.6 Hz), 5.01 (m, 1H), 5.01 (d, 1H, *J* = 17.5 Hz), 5.16 (d, 1H, *J* = 10.9 Hz), 5.79 (dd, 1H, *J* = 10.9, 17.5 Hz); ^13^C-NMR (125 MHz) δ 17.5, 17.8, 20.0, 20.4, 20.9, 22.3, 25.6, 26.5, 32.9, 38.6, 38.7, 43.3, 44.5, 47.7 (2C), 52.2, 53.3, 63.0, 65.6, 114.5, 124.1, 131.5, 143.3, 171.0, 172.7; HRMS calcd for C_25_H_39_NO_5_S (M^+^) *m/z* 465.2549, found 465.2556.

*(2R)-N-[(2S,3R)-2-(Acetoxymethyl)-3,7-dimethyl-3-vinyloct-6-enoyl]bornane-10,2-sultam* (**19b**). As described for the preparation of **19a** from **7a**, compound **7b** (33.7 mg, 79.9 μmol) was treated with NaBH_4_ (1.5 mg, 40 μmol) in EtOH (1 mL) to provide crude alcohol (37.0 mg), which was then treated with Ac_2_O (19 μL, 0.20 mmol), Et_3_N (33 μL, 0.24 mmol), and DMAP (1.1 mg, 9.0 μmol) in CH_2_Cl_2_ (1 mL) to provide 24.2 mg (65% for 2 steps) of **19b** as a colorless oil: TLC *R_f_* 0.68 (EtOAc/hexane, 1:2); [α]_D_^20^–54.7 (*c* 1.06, CHCl_3_); IR (neat) 2966, 2886, 1742, 1687 cm^−1^; ^1^H-NMR (500 MHz) δ 0.97 (s, 3H), 1.18 (s, 3H), 1.19 (s, 3H), 1.35–1.48 (m, 3H), 1.57 (br s, 3H), 1.61 (m, 1H), 1.65 (br s, 3H), 1.86–1.93 (m, 5H), 1.95 (s, 3H), 2.10 (dd, 1H, *J* = 7.8, 13.8 Hz), 2.19 (m, 1H), 3.26 (dd, 1H, *J* = 3.7, 10.6 Hz), 3.47 (d, 1H, *J* = 13.8 Hz), 3.52 (d, 1H, *J* = 13.8 Hz), 3.94 (dd, 1H, *J* = 5.2, 7.8 Hz), 4.06 (t, 1H, *J* = 10.6 Hz), 4.56 (dd, 1H, *J* = 3.7, 10.6 Hz), 5.02 (d, 1H, *J* = 17.4 Hz), 5.06 (m, 1H), 5.16 (d, 1H, *J* = 11.3 Hz), 5.84 (dd, 1H, *J* = 11.3, 17.4 Hz); ^13^C-NMR (125 MHz) δ 17.6, 18.6, 19.9, 20.8, 21.1, 22.5, 25.7, 26.3, 33.0, 37.9, 38.6, 42.4, 44.6, 47.7, 47.8, 52.0, 53.3, 64.6, 65.8, 114.2, 124.5, 131.2, 143.9, 170.6, 172.6; HRMS calcd for C_25_H_39_NO_5_S (M^+^) *m/z* 465.2549, found 465.2558.

*(2R)-N-[(2R,3S)-2-(Acetoxymethyl)-6-(tert-butyldiphenysilyloxy)-3-methyl-3-vinylhexanoyl]bornane-10,2-sultam* (**20a**). As described for the preparation of **19a** from **7a**, compound **15a** (150 mg, 236 μmol) was treated with NaBH_4_ (4.4 mg, 0.12 mmol) in EtOH (3 mL) to provide crude alcohol (152 mg), which was then treated with Ac_2_O (56 μL, 0.59 mmol), Et_3_N (99 μL, 0.71 mmol), and DMAP (3.0 mg, 25 μmol) in CH_2_Cl_2_ (3 mL) to provide 151 mg (94% for 2 steps) of **20a** as a colorless oil: TLC *R_f_* 0.66 (EtOAc/toluene, 1:5); [α]_D_^23^–28.6 (*c* 2.01, CHCl_3_); IR (neat) 2960, 2859, 1744, 1691 cm^−1^; ^1^H-NMR (500 MHz) δ 0.92 (s, 3H), 1.03 (s, 9H), 1.03 (s, 3H), 1.07 (s, 3H), 1.32–1.46 (m, 5H), 1.72 (m, 1H), 1.80 (m, 1H), 1.88–1.90 (m, 2H), 1.98 (s, 3H), 2.06–2.09 (m, 2H), 3.34 (m, 1H), 3.41 (d, 1H, *J* = 13.8 Hz), 3.47 (d, 1H, *J* = 13.8 Hz), 3.54–3.60 (m, 2H), 3.92 (t, 1H, *J* = 6.3 Hz), 4.22 (t, 1H, *J* = 10.6 Hz), 4.36 (dd, 1H, *J* = 3.4, 10.6 Hz), 4.99 (d, 1H, *J* = 17.5 Hz), 5.13 (d, 1H, *J* = 11.0 Hz), 5.75 (dd, 1H, *J* = 11.0, 17.5 Hz), 7.35–7.43 (m, 6H), 7.63-7.64 (m, 4H); ^13^C-NMR (125 MHz) δ 18.2, 19.1, 19.9, 20.5, 20.9, 26.5, 26.8 (3C), 27.0, 32.9, 34.4, 38.6, 43.0, 44.4, 47.6 (2C), 52.2, 53.3, 63.0, 64.1, 65.6, 114.6, 127.6 (4C), 129.5 (2C), 133.9, 134.0, 135.5 (2C), 135.6 (2C), 143.2, 171.0, 172.6; HRMS calcd for C_34_H_44_NO_6_SSi (M^+^–*t*-C_4_H_9_) *m/z* 622.2659, found 622.2677.

*Synthesis of* **19a** *from* **20a**. To a cooled (0 °C) stirred solution of **20a** (12.2 mg, 17.9 μmol) in THF (3 mL) was added HF·pyridine (0.2 mL). The mixture was stirred at room temperature for 5 h and quenched with saturated aqueous NaHCO_3_ (1 mL). This was diluted with H_2_O (15 mL) and extracted with CH_2_Cl_2_ (10 mL × 4). The combined extracts were dried and concentrated under reduced pressure. The residue was purified by column chromatography on silica gel (EtOAc/hexane, 1:2) to provide 8.0 mg (quant.) of alcohol as white crystals: mp 113-115 °C; TLC *R_f_* 0.24 (EtOAc/hexane, 1:2); [α]_D_^20^ –42.7 (*c* 1.02, CHCl_3_); IR (neat) 3529, 2961, 2882, 1741, 1690 cm^−1^; ^1^H-NMR (500 MHz) δ 0.97 (s, 3H), 1.09 (s, 3H), 1.16 (s, 3H), 1.35–1.58 (m, 5H), 1.67 (m, 1H), 1.88-1.92 (m, 3H), 1.99 (s, 3H), 2.09–2.15 (m, 2H), 3.37 (m, 1H), 3.44 (d, 1H, *J* = 13.9 Hz), 3.50 (d, 1H, *J* = 13.9 Hz), 3.54 (m, 1H), 3.61 (m, 1H), 3.95 (t, 1H, *J* = 6.5 Hz), 4.20 (t, 1H, *J* = 10.7 Hz), 4.40 (dd, 1H, *J* = 3.4, 10.7 Hz), 5.02 (d, 1H, *J* = 17.5 Hz), 5.15 (d, 1H, *J* = 11.3 Hz), 5.80 (dd, 1H, *J* = 11.3, 17.5 Hz); ^13^C-NMR (125 MHz) δ 18.7, 19.9, 20.5, 20.9, 26.5, 27.1, 32.9, 34.4, 38.6, 42.9, 44.5, 47.7 (2C), 51.8, 53.3, 63.0, 63.1, 65.7, 114.5, 143.1, 171.2, 172.6; HRMS calcd for C_22_H_35_NO_6_S (M^+^) *m/z* 441.2185, found 441.2192.

To a cooled (0 °C) stirred solution of alcohol (20.9 mg, 47.3 μmol) in CH_2_Cl_2_ (1 mL) was added Dess–Martin periodinane (30.3 mg, 71.4 μmol). The mixture was stirred at room temperature for 2 h and Dess–Martin periodinane (31.1 mg, 73.3 μmol) was added. After being stirred at room temperature for 2.5 h, the mixture was quenched with saturated aqueous Na_2_S_2_O_3_ (3 mL) and saturated aqueous NaHCO_3_ (3 mL), diluted with H_2_O (4 mL), and extracted with CH_2_Cl_2_ (15 mL × 3). The combined extracts were washed with saturated brine (20 mL), dried and concentrated under reduced pressure. The residue was purified by column chromatography on silica gel (EtOAc/hexane, 1:7) to provide 20.8 mg (quant.) of aldehyde as a colorless oil, which was immediately used in the next step: TLC *R_f_* 0.33 (EtOAc/hexane, 1:2); ^1^H-NMR (300 MHz) δ 0.97 (s, 3H), 1.07 (s, 3H), 1.17 (s, 3H), 1.33–1.48 (m, 4H), 1.68 (m, 1H), 1.89–2.04 (m, 2H), 1.99 (s, 3H), 2.11–2.13 (m, 2H), 2.41 (t, 2H, *J* = 7.8 Hz), 3.38 (m, 1H), 3.44 (d, 1H, *J* = 13.9 Hz), 3.52 (d, 1H, *J* = 13.9 Hz), 3.95 (t, 1H, *J* = 6.5 Hz), 4.21 (t, 1H, *J* = 10.7 Hz), 4.37 (dd, 1H, *J* = 3.6, 10.7 Hz), 5.05 (d, 1H, *J* = 17.5 Hz), 5.20 (d, 1H, *J* = 10.7 Hz), 5.78 (dd, 1H, *J* = 10.7, 17.5 Hz), 9.73 (s, 1H).

The following reaction was carried out under Ar. To a cooled (0 °C) stirred suspension of *i*-PrP^+^Ph_3_I^−^ (21.9 mg, 49.1 μmol) in THF (1 mL) was added *t*-BuLi (1.61 M solution in pentane, 29 μL, 47 μmol). The mixture was stirred at 0 °C for 30 min and a solution of aldehyde (6.9 mg, 16 μmol) in THF (1 mL) was added. After being stirred at 0 °C for 20 min, the mixture was diluted with H_2_O (10 mL) and extracted with CH_2_Cl_2_ (15 mL × 3). The combined extracts were dried and concentrated under reduced pressure. The residue was purified by column chromatography on silica gel (EtOAc/hexane, 1:15) to provide 3.3 mg (45%) of **19a**.

*(2R)-N-[(2R,3S)-2-(Acetoxymethyl)-5-(1,3-dioxolan-2-yl)-3-methyl-3-vinylpentanoyl]bornane-10,2-sultam* (**21a**). As described for the preparation of **19a** from **7a**, compound **18a** (154 mg, 350 μmol) was treated with NaBH_4_ (6.5 mg, 0.17 mmol) in EtOH (3 mL) to provide crude alcohol (158 mg), which was then treated with Ac_2_O (83 μL, 0.88 mmol), Et_3_N (146 μL, 1.05 mmol), and DMAP (4.4 mg, 36 μmol) in CH_2_Cl_2_ (4 mL) to provide 150 mg (88% for 2 steps) of **21a** as a colorless oil: TLC *R_f_* 0.65 (EtOAc/toluene, 1:2); [α]_D_^21^–39.5 (*c* 1.02, CHCl_3_); IR (neat) 2962, 2884, 1743, 1690 cm^−1^; ^1^H-NMR (500 MHz) δ 0.97 (s, 3H), 1.08 (s, 3H), 1.16 (s, 3H), 1.36 (m, 1H), 1.43–1.50 (m, 2H), 1.55–1.60 (m, 2H), 1.78 (m, 1H), 1.87–1.91 (m, 3H), 1.99 (s, 3H), 2.11–2.18 (m, 2H), 3.35 (m, 1H), 3.43 (d, 1H, *J* = 13.7 Hz), 3.50 (d, 1H, *J* = 13.7 Hz), 3.78–3.81 (m, 2H), 3.91–3.95 (m, 3H), 4.22 (t, 1H, *J* = 10.7 Hz), 4.38 (dd, 1H, *J* = 3.5, 10.7 Hz), 4.76 (t, 1H, *J* = 4.6 Hz), 5.01 (d, 1H, *J* = 17.5 Hz), 5.17 (d, 1H, *J* = 10.7 Hz), 5.77 (dd, 1H, *J* = 10.7, 17.5 Hz); ^13^C-NMR (125 MHz) δ 18.1, 20.0, 20.5, 20.9, 26.5, 28.3, 32.3, 32.9, 38.6, 42.8, 44.5, 47.7 (2C), 52.2, 53.3, 63.0, 64.7, 64.8, 65.6, 104.5, 114.9, 142.9, 171.0, 172.5; HRMS calcd for C_24_H_37_NO_7_S (M^+^) *m/z* 483.2291, found 483.2291.

*Synthesis of* **19a** *from* **21a**. A solution of **21a** (80.5 mg, 166 μmol) in THF (12 mL) and 4 M aqueous HCl (12 mL) was stirred at 0 °C for 15 h, diluted with saturated aqueous NaHCO_3_ (50 mL), and extracted with CH_2_Cl_2_ (60 mL × 3). The combined extracts were dried and concentrated under reduced pressure. The residue was purified by column chromatography on silica gel (EtOAc/hexane, 1:7) to provide 58.2 mg (80%) of aldehyde, which was identical with the aldehyde derived from **20a** and converted into **19a** as described above.

## 4. Conclusions

In conclusion, we have developed an asymmetric Claisen rearrangement using Oppolzer’s camphorsultam as a chiral auxiliary. Notably, rearrangement products **7a**, **15a**, and **18a** possess a chiral quaternary carbon with high enantiomeric purity. In addition, this method has been applied to the total synthesis of (+)-bakuchiol (**4**). Further studies and applications of this work to natural product synthesis are in progress and will be reported in due course.
